# A contribution to knowledge on the terrestrial malacofauna of the Kastellorizo (Megisti) island group (SE Greece)

**DOI:** 10.1186/s40709-019-0107-9

**Published:** 2019-11-08

**Authors:** Moisis Mylonas, Katerina Vardinoyannis, Nikos Poulakakis

**Affiliations:** 1 Natural History Museum of Crete, School of Sciences and Engineering, University of Crete, 71409 Irakleio, Greece; 2 Biology Department, School of Sciences and Engineering, University of Crete, Voutes University Campus, 70013 Irakleio, Greece

**Keywords:** Biodiversity, Land Gastropods, Taxonomy, Ecology, Biogeography

## Abstract

**Background:**

The Kastellorizo island group (in the Dodecanese, Greece) is situated in the southeast corner of the Aegean Archipelago. It consists of twenty islets, of which the three largest (Kastellorizo, Ro and Strongyli) and seven smaller ones belong to Greece. Knowledge of the malacofauna on the islands is relatively poor. Only eight species were known prior to the present study, all from the islet of Kastellorizo.

**Results:**

Here, using the scientific collections at the Natural History Museum of Crete collected mainly by the authors and also by several researchers since 1976, we reappraise the malacofauna of the island group. Thirty-one species were found in total (23 from Kastellorizo, 19 from Ro, 15 from Strongyli, 10 from Agios Georgios, 14 from Agrielia, 6 from Psomi and 10 from Psoradia).

**Conclusions:**

The fact that there are no endemic snail species in the islands can be accounted for by their proximity to the Turkish coast, their common paleogeography with Turkey until the Late Pleistocene and Holocene, and the influence of humans. All but two species, *Mastus etuberculatus* and *Vitrea riedeliana*, are known from the adjacent Turkish coasts. Together with the subfossil species found on the smaller islets, the predominance of different species on each islet suggests a continuous substitution from the source areas of Turkey and the Aegean.

## Background

**Fig. 1:**
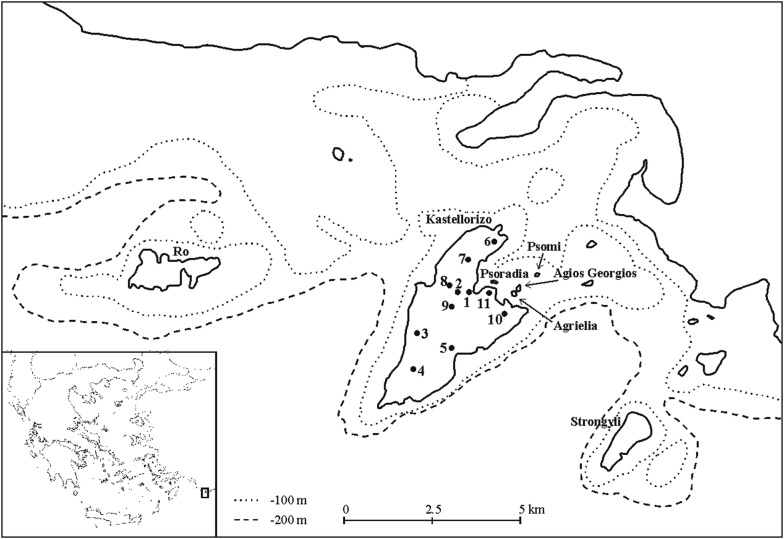
The studied islets and the sampling sites on Kastellorizo

The Kastellorizo (=Megisti) island group is situated in the southeast corner of the Aegean Archipelago, 140 km east of Rhodes and less than 5 km south of the Turkish coast (Fig. 1). It consists of 20 islands, half of which belong to Greece and the other half to Turkey. Of the largest ones, Kastellorizo, Ro and Strongyli, belong to Greece. Kastellorizo is the only inhabited and accessible island, while special permission is required to visit the others.

All the islands are formed of limestone and sediments. Calcareous cliffs and rocks exist on the biggest islets. The climate is thermomediterranean [1], and the predominant vegetation consists of phrygana and maquis. Human presence has been intensive and continuous for over 2000 years on Kastellorizo alone, which was historically one of the safest ports in the Eastern Mediterranean basin. The few hundred present-day inhabitants work in tourism, agriculture and livestock farming. Due to their environmental characteristics, all the Greek islands in the Kastellorizo group have been placed within the “Natura 2000” network.

Our knowledge on the terrestrial malacofauna of the island group was very poor, as the extremely limited previous data were restricted to the island of Kastellorizo. H Rolle was the first person to collect land snails there in 1894, followed almost a century later by A Liebegott in 1983 and 1996. A total of eight species were reported, and all references are commented in the discussion.

This paper presents an extensive study of the local malacofauna, based on a wealth of material collected by the authors in 1996, as well as on samples occasionally gathered by other scientists since 1976 and deposited in the collections of the Natural History Museum of Crete—University of Crete (NHMC). Taxonomical, ecological and distributional characteristics of the species found are also discussed in relation to the malacofauna of nearby areas and other islands in the Aegean.

## Results

In total, 31 species of land snails and slugs were found on the seven islets studied (Table 1).

**Table 1:** Species found on the island group of Kastellorizo

Island		Kastellorizo											Ro	Strongyli	Agios Georgios	Agrielia	Psomi	Psoradia
Species		1	2	3	4	5	6	7	8	9	10	11						
1. * *Helix asemnis* Bourguignat, 1860		+	+	+	+	+	+	+	+	+	+	+		ex				
2.* *Helix nucula* Mousson, 1854		+											+	ex	+	+		
3. *Cantareus apertus* Born, 1778													+					
4. * *Isaurica lycia* (Martens, 1889)		+	+				+	+		+	+							
5. *Eobania vermiculata* (Müller, 1774)		+											+	+	+	+	+	+
6. * *Metafruticicola rugosissima* Bank, Gittenberger, Neubert, 2013		+	+	+	+	+	+	+	+	+	+	+		+	+	+	+	+
7. *Metafruticicola schuberti* (Roth, 1839)													+					
8. *Caracollina lenticula* (Férussac, 1821)		+										+	+	+	+		+	+
9. *Cochlicella acuta* (Müller, 1774)		+											+					
10. *Cernuella virgata* (Da Costa, 1778)		+											+					
11. *Xeropicta krynickii* (Krynicki, 1833)													+					+
12. *Zebrina fasciolata* (Olivier, 1801)													sh					
13. *Mastus rossmaessleri* (L. Pfeiffer, 1847)						+		+							+	+		
14. *Mastus etuberculatus* (Frauenfeld, 1867)														+				
15. * *Albinaria anatolica* (Roth, 1878		+	+	+	+	+	+	+	+	+	+	+	+	+	+	+		+
16. * Rumina saharica* (Pallary, 1901)		+	+				+	+	+				+					ex
17. *Pleurodiscus balmei* (Potiez & Michaud, 1838)		+					+	+	+						+	+		
18. * *Zonites caricus* (Roth, 1839)		+	+	+	+	+	+	+	+	+	+	+	ex	ex		ex		ex
19. * Eopolita protensa* (Férussac, 1832)		+																
20. *Schistophallus cyprius* (L. Pfeiffer, 1847)		+							+				+		+	+		+
21. *Mediterranea hydatina* (Rossmässler, 1838)		+																
22. *Vitrea contracta* (Rossmässler, 1842)		+		+									+			+		
23. *Vitrea riedeliana* Paget, 1976																+		+
24. *Truncatellina rothi* (Reinhardt, 1916)				+									+	+			+	
25. *Granopupa granum* (Draparnaud, 1801)		+							+	+				+		+		
26. *Rupestrella rhodia* (Roth, 1839)		+	+	+	+	+	+	+		+			+	+		+		
27. *Orculella ignorata* Hausdorf, 1996														+	+	+	+	
28. *Cecilioides michoniana* (Bourguignat, 1864)						+	+	+					+	+			+	+
29. *Cecilioides tumulorum* (Bourguignat, 1864)						+		+						+				
30. *Tandonia pageti* (Forcart, 1972)		+		+	+	+	+	+	+	+	+		+	+				
31. *Deroceras rethimnonense* de Winter & Butot, 1986													+		+	+		
Total number of extant species		19	7	8	6	9	10	12	9	8	6	5	17	12	10	13	6	8

+: Extant species on site or island; ex: extinct; sh: one fresh shell; 1–11: sites on Kastellorizo Isl. *Asterisks in numbers depict the species that have been referred from Kastellorizo Isl

The majority (23 species) occurs on Kastellorizo, which is the largest island. Nineteen species were collected at a single site, in and around the only village, among ruined houses, in yards and small gardens - this is by far the richest site on the island. One of the above species, *Eopolita protensa*, was confirmed on the basis of a single fresh adult shell found by herpetologists M Polymeni and E Valakos in 1976.

Six of the species found on Kastellorizo (*Helix asemnis, Metafruticicola rugosissima, Albinaria anatolica, Zonites caricus, Rupestrella rhodia* and *Tandonia pageti*) comprise the basic core of the malacological diversity of the island, being found in dense populations in all or in most (> 8) sites. By contrast, seven other species (*Helix nucula, Eobania vermiculata, Cochlicella acuta, Cernuella virgata, Mediterranea hydatina, Eopolita protensa* and *Monacha rothi*) can be characterized as rare, as only few individuals were found at a single site.

Nineteen species were found on Ro, the second largest islet. Dense populations of two of them (*Cantareus apertus* and *Metafruticicola schuberti*) were found here, but were absent from all the other islets. A single shell of *Zebrina fasciolata* was found on the north coast of the islet, and *Zonites caricus* was found only as a subfossil in several localities.

Fifteen species occur on Strongyli, the third largest islet. Three of these (*Helix asemnis, Helix nucula* and *Zonites caricus*) were only found as subfossils. The species *Mastus etuberculatus* was seen here alone, in extremely dense populations.

Although the islet of Agrielia is very small (0.012 km^2^) we collected 14 species, all extant except *Zonites caricus*, which was found only as a subfossil.

Malacofauna on the three remaining islets, namely Agios Georgios, Psoradia and Psomi, consists of 10, 10 and 6 species, respectively. On Psoradia islet only subfossils shells of *Rumina saharica* and *Zonites caricus* were found.

*Taxonomical, ecological and distributional remarks.*

1. *Helix (Helix) asemnis* and *Helix (Pelasga) nucula* (Fig. 2a, b): The two species can be distinguished on the basis of both shell and genitalia. *Helix asemnis* has a larger shell and short epiphallus, while *H. nucula* has a smaller shell and a very long epiphallus [2]. *Helix asemnis* is very common and was encountered in dense populations on Kastellorizo, whereas on Strongyli islet it was found only as a subfossil. *Helix nucula* dominates on Ro Island; it was also found extant on the islets of Agios Georgios and Agrielia, but only as a subfossil on Strongyli. Very few fresh shells of the species were found around the village on Kastellorizo.

**Fig. 2:**
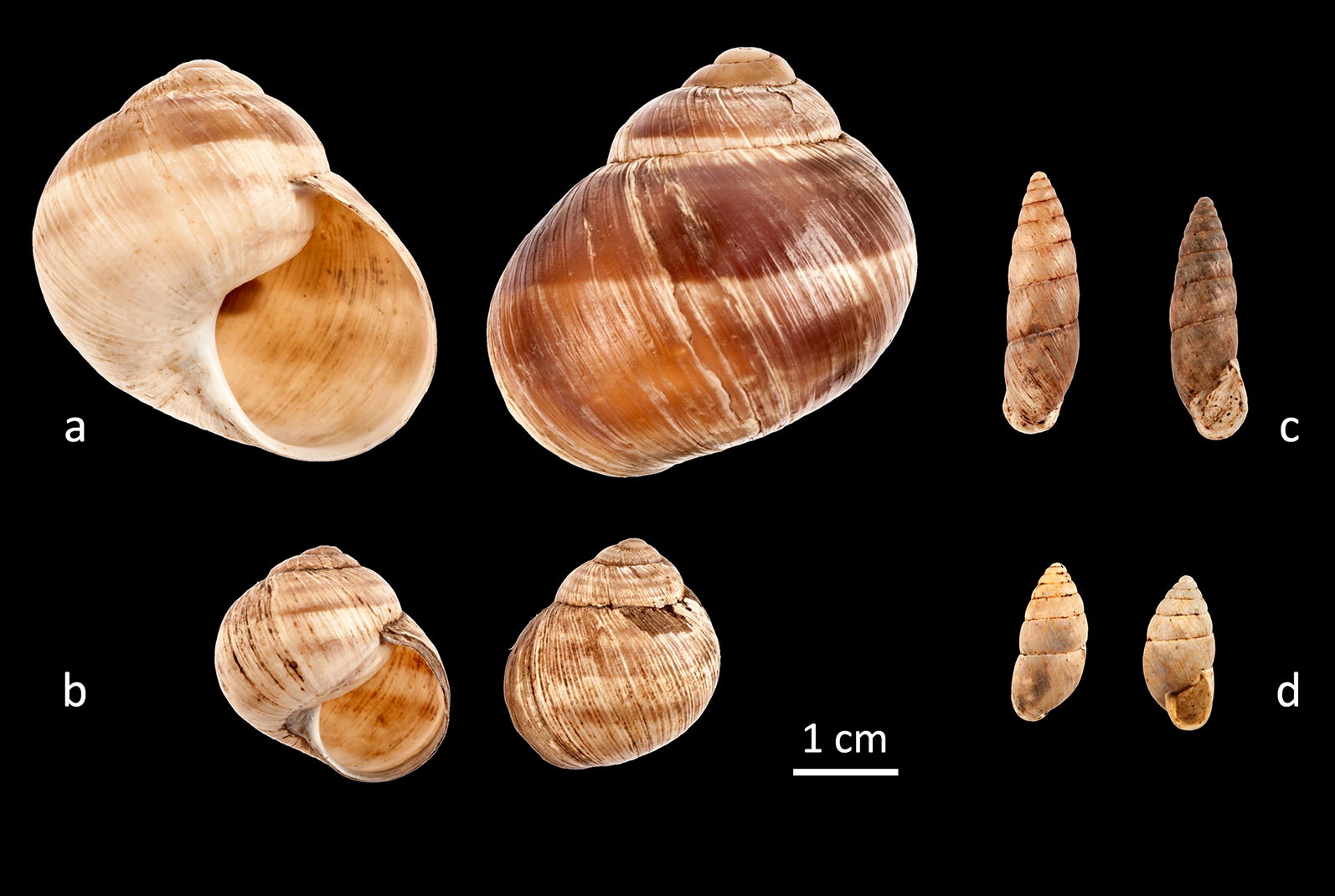
Shells of **a** *Helix asemnis*; **b** *Helix nucula*; **c** *Mastus rossmaessleri*; **d** *Mastus etuberculatus*
2. *Isaurica lycia*: This rock dwelling species is common on Kastellorizo, with dense populations in rocky habitats and on cliffs, but is absent from all the other islets. According to Neubert et al. [3] it is very common on the adjacent coast around Kas.
3. *Zebrina fasciolata*: One fresh shell was found on the island of Ro, close to the seashore. This was probably an accidental appearance, though the species has not been reported on the coast of Kas [3].
4. *Mastus rossmaessleri* and *Mastus etuberculatus* (Fig. 2c, d): *M. rossmaessleri* was found in two localities on Kastellorizo and the nearby islets of Agios Georgios and Agrielia, though the species is very common throughout the region of Kas and Demre [3]. *Mastus etuberculatus* was only found on the island of Strongyli, where it forms dense populations. This species has a wide distribution, from the central Aegean islands to the southwestern coasts of Turkey [4], but is absent from the nearby mainland. The two species are easily recognized by their shell height (SH), shell width (SD) and number of whorls (R) (*M. rossmaessleri*: SH = 16.5–22.6 mm, SD = 6.1–6.7 mm, R = 7.5–8.5, *M. etuberculatus*: SH = 13.2–14.8 mm, SD = 6.2–6.9, R = 6.5–7).
5. *Eobania vermiculata*: Although this is one of the commonest species throughout the Eastern Mediterranean and very synanthropic, only one sparse population was found around the village on Kastellorizo, which is most affected by man, whereas dense populations occur on all the other islets in the group. It is not common in the nearby areas of Kas and Demre: of 67 sampling localities mentioned by Neubert et al. [3], only one population was found close to Demre.
6. *Metafruticicola rugosissima* and *Metafruticicola schuberti* (Fig. 3). The species *M. rugosissima* was described by Bank et al. [5], with Kastellorizo being the type locality of the species. Welter-Schultes [6] was the first to mention it as a new species, though without naming it. Dense populations are also distributed in nearby areas of Turkey [6]. We observed dense populations of this species everywhere on Kastellorizo and the remaining islets in our study except for Ro, where we found *M. schuberti*. According to Bank et al. [5], the latter is distributed in south Turkey, where it was found sympatric with *M. rugosissima* in some localities, without hybrids. Further to the shell differences [5] we examined the genital system of the two species for the first time. We looked at 12 specimens of *M. rugosissima* from Kastellorizo and Strongyli and 6 specimens of *M. schuberti* from Ro islet. The two species clearly differ as regards the form of the penis papillae, the length and diameter of the vagina, which is longer and thicker in *M. rugosissima*, as well as the beginning of the spermatheca duct, which is broader in *M. schuberti*. The main characteristics of the genitalia of *M. schuberti* from Ro islet resemble those of *M. hikmeti* given by Hudec [7], which Bank et al. [5] considered a synonym of *M. schuberti* on the basis of shell morphology.

**Fig. 3:**
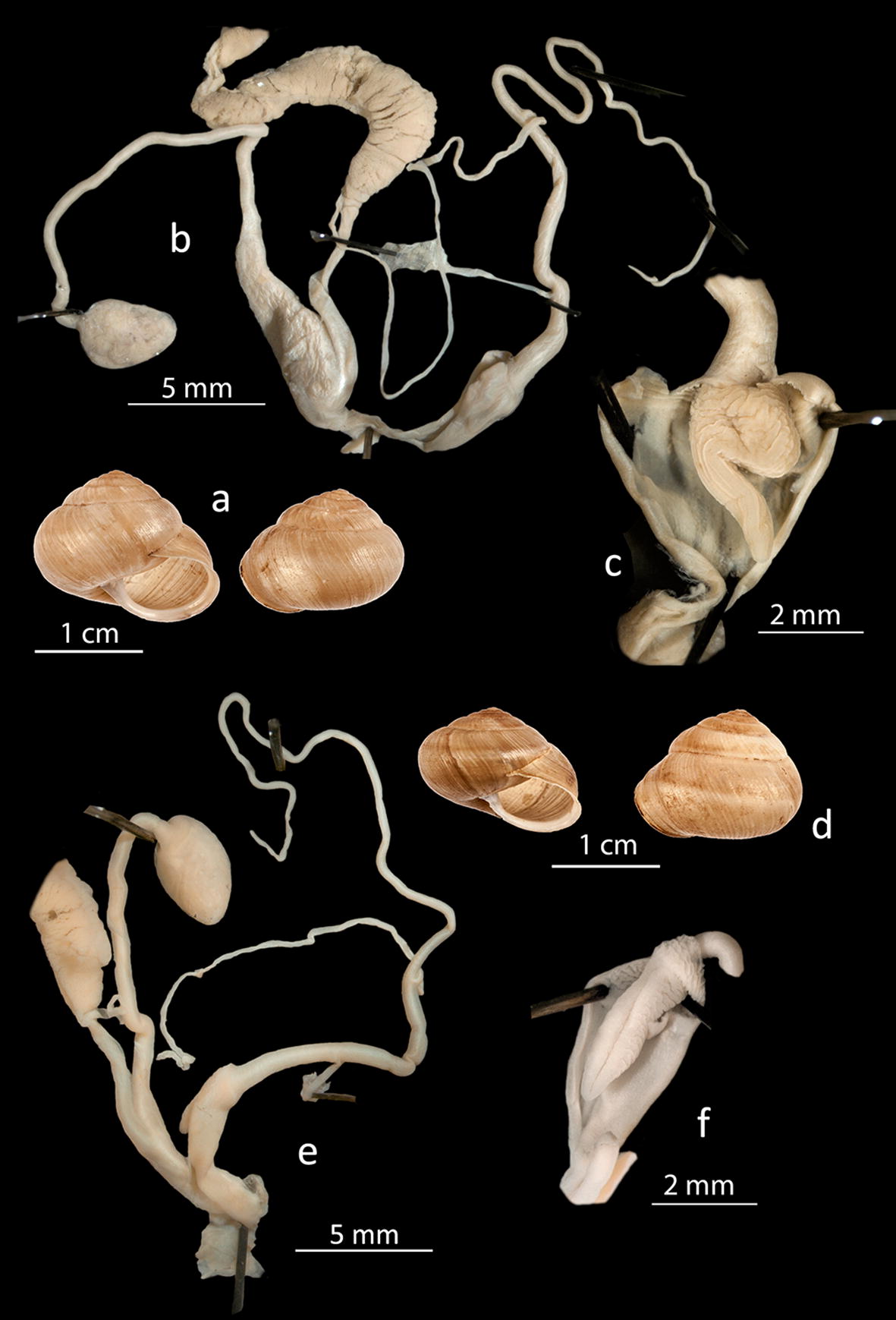
Shell and genitalia of *Metafruticicola schuberti* (**a**–**c**) and *Metafruticola rugosissima* (**d**–**f**). *M. schuberti* a: shell; **b** genitalia; **c** penis papilla. *M. rugosissima,* **d** shell; **e** genitalia; **f** penis papilla
7. *Vitrea riedeliana* and *Vitrea contracta* (Fig. 4c, d). *Vitrea riedeliana* is commonly found in dense populations on Rhodes [8] and the nearby coasts of Turkey [4]. Discovery of the species on the islets of Agrielia and Psoradia could be attributed either to accidental introduction or to a wider unknown distribution. On the other hand, the common palearctic *V. contracta* was found at two sites on Kastellorizo, and also on Ro and Agrielia, where it lives sympatric with *V. riedeliana*. Dense populations were only found in the village on Kastellorizo.

**Fig. 4:**
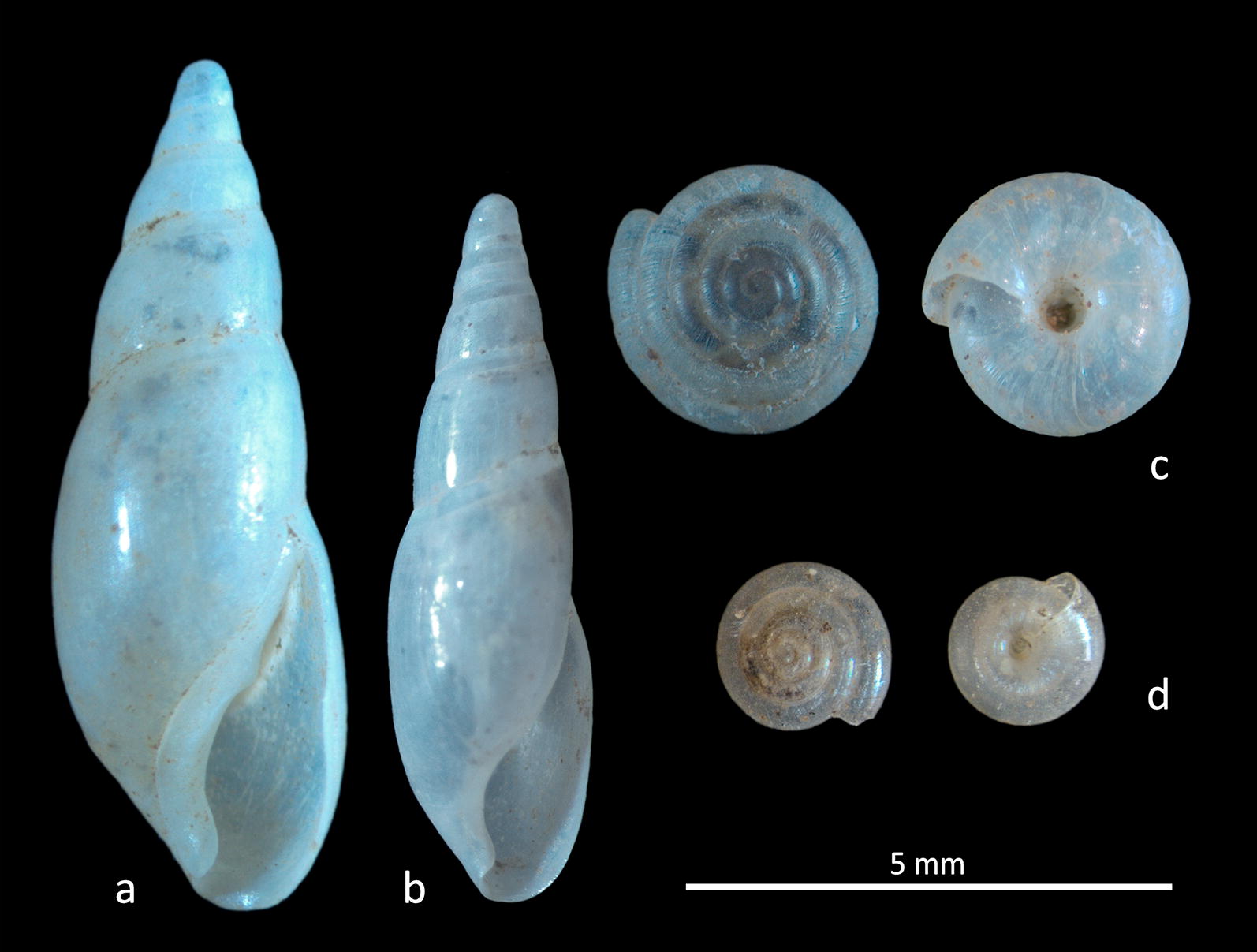
Shells of **a** *Cecilioides michoniana*; **b** *Cecilioides tumulorum*; **c** *Vitrea riedeliana*; **d** *Vitrea contracta*
8. *Cecilioides michoniana* and *C. tumulorum* (Fig. 4a, b). Species classification within the genus *Cecilioides* is still highly problematic, especially in the Eastern Mediterranean [9, 10]. Three species in the genus are common on the opposite coasts of Turkey: *C. acicula, C. tumulorum* and *C. michoniana* [3]. In the Kastellorizo Island group dense populations were only found only on Strongyli. The specimens collected there were classified as *C. tumulorum* (SH = 5.5–6.8 mm, SD = 1.8–2.1 mm, R = 6–7.2) and *C. michoniana* (SH = 7.5–8.3 mm, SD = 2.8–3 mm, R = 6.2–6.8), which predominate. Whether sympatric or not, these species were also found on other studied islets, though always in sparse populations. *Cecilioides tumulorum* is shorter and slender and has a smaller aperture than *C. michoniana.* Fully grown individuals of the latter, larger than 7.5 mm, have a subtle callus on the parietal lip.

## Discussion

Prior to this study, our knowledge concerning the malacofauna of the Kastellorizo island group was restricted to eight species, all recorded from Kastellorizo Island itself. Two of these species were not found in this survey: *Levantina spiriplana*, mentioned by Zilch [11] and Mienis [12], and *Buliminus carneus*, mentioned by Rolle and Kobelt [13], Böttger (as *B. carneus f. minor*) [14], Forcart (as *Petraeus carneus glabratus*) [15], and Bank and Hovestadt [16]. The relatively large shells of both species in relation to our intense survey and their absence from the adjacent mainland lead us to conclude that they should not be included in the terrestrial malacofauna of Kastellorizo Island. With the exception of *Helix nucula*, the remaining six species are very common in and around the village of Kastellorizo. *Albinaria anatolica* was mentioned by Pfeiffer [17], Paget [18], Nordsieck [19], Giokas [20] and Schütt [21]. *Helix asemnis* was first reported from Kastellorizo by Knipper [22] (as *Helix venusta*) and later by Schütt [23]. When studying the Liebegott collections, Neubert [2] classified it as *H. asemnis,* and also cites the species *H. nucula* in the same work. Pfeiffer and Wachter [24] and later Zilch [11] recorded the species *Isaurica lycica*. Based on the same material, Subai [25] classified it as *I. lycia. Zonites caricus* was first reported from Kastellorizo by Rolle (as *Z. megistus*) [26] and by Pfeiffer (as *Z. lycicus*) [27]; later Zilch [28] classified it as *Z. caricus*, and since then this name was used by various scientists [3, 6, 8, 23, 29–31]. *Metafruticicola rugosissima* was described as a new species by Bank et al. [5], based on material collected by Liebegott. Earlier on, Neubert et al. [3] and Welter Schultes [6, 32] had pointed out the particularities of the species without naming it.

Of all the seven islands in this study, the richest in terrestrial molluscs is Kastellorizo, with 23 species. An astonishing 19 species - 86% of all malacofauna on the island - were found around the only village there. This is the highest percentage ever found in and around the main village and/or port of an Aegean island; comparable figures for Cycladic sites range between 55 and 75% [33]. This exceptionally high number of species could be attributed to continuous settlement of the site for over 2500 years, including periods when it was a small town of 14,000 people, in contrast to the 220 people that live on the island at the present time. Ro Island, with restricted human influence in a handful of cultivated fields, has seventeen living species, whilst on Strongyli, where the only human influence is livestock breeding, there are twelve. In proportion to its area of 0.01 km^2^, the islet of Agrielia has an exceptionally high number of living species (13 in total). This may be attributable to the fact that it is only 200 m from the village of Kastellorizo.

The cluster analysis was not informative, since clustering was very loose. When analysis was carried out with Jaccard similarity index it appears that the richest site of Kastellorizo (site 1) grouped with all the other islets, while the remaining sites clustered altogether. In all the analyses the clusters did not reflect any relation with area, habitat or proximity.

The predominant species on the three largest islets differ both quantitatively and qualitatively. Six species constitute the core of the malacofauna on Kastellorizo, with *Zonites caricus* and *Metafruticicola rugosissima* being the commonest. On Ro Island, the commonest are *E. vermiculata*, *C*. *apertus* and *Metafruticicola schuberti*, while on Strongyli, they are *Caracollina lenticula*, *Metafruticicola rugosissima*, *Mastus etuberculatus* and *Cecilioides michoniana.* This phenomenon was also observed in the islets around Kalymnos [34] and of the Argolic and Saronic gulf [35] and was attributed to a continuous substitution from the nearby mainland, Turkey and Greece respectively.

Comparing the diversity of this island group with other islands of similar area in the Aegean, from the Kalymnos, Astypalaia and Skyros groups [34] and the islands in the Argolic and Saronic Gulfs [35], the diversity of the Kastellorizo group would appear to be poor. It is as low as that found on the satellite islets of Crete, which are of similar size [36]. In the latter case, however, distances from Crete are much greater and isolation higher. The reasons for this poverty in the Kastellorizo group are far from obvious. Neither isolation nor recent unfavorable ecological conditions can be responsible, considering that Constantinidis [37] found 477 plant species on Kastellorizo group flora survey, rendering it far richer than that of Aegean islands with similar area/s.

Of the 31 species found, *Zebrina fasciolata* cannot be considered native to the Kastellorizo group, as shell appearance there was probably accidental. Four species with living populations in Kastellorizo are found as subfossils on the smaller islands. These differences in extant and extinct species could be related to a reduction in the islets’ area after the last glacial maximum, and to the dispersal ability of many species from the source areas of Turkey and the Aegean.

Although the paleogeography of the Kastellorizo island group remains unknown, if we take into consideration the isobaths of the area and a decrease in sea level of at least 125 m during the Würm glacial period (20,000–15,000 years ago) [38], we can assume that the three larger islands of Kastellorizo, Ro and Strongyli were not directly connected, forming three distinct peninsulas of the nearby mainland (Fig. 1). This assumption is further supported by the different characteristic species found on the three islets in question.

Neubert et al. [3] recorded 32 species of land gastropods on the adjacent mainland in an area measuring 400 km^2^ between Kas and Demre in southwest Turkey, whereas our study brought 31 species to light on seven islets with a total area of less than 13 km^2^. It is obvious that many more species are to be found in the above area of mainland Turkey. Of the 31 species found in the present study, only *Mastus etuberculatus* and *Vitrea riedeliana* have not been reported on the nearby area of Turkey.

## Methods

From 2 to 10 December 1996 the authors collected a large number of land snails, as part of a scientific expedition by the Natural History Museum of Crete to the Greek islands in the Kastellorizo island group. The collecting effort was based on the size, geomorphology and vegetation of each islet (Table 2), sampling in all different types of habitats. Land snails were thus collected from 11 localities on the largest islet (Kastellorizo). Samples were collected from Ro and Strongyli during full-day excursions, while 3–4 h were spent on each of the smallest islets (Agios Georgios, Agrielia, Psomi and Psoradia). The type of vegetation and the influence of man were recorded at each sampling site, and litter was collected from the predominant plants.

**Table 2:** Characteristics of islands and sites of Kastellorizo islet group

Islands	Area (km^2^)	Altitude (m)	Habitats
Kastellorizo isl.	9.78	273	
Site 1, Kastellorizo in and around the village			Cultivations, ruins, pinewood, phrygana, calcareous rocks
Site 2, Kastellorizo 1 km west of the village, north of Vigla Mt.			Phrygana, calcareous rocks
Site 3, Southern part of the island, west of the airport			Phrygana
Site 4, Southern part of the island			Maquis, phrygana, calcareous rocks
Site 5, Navlakas around			Phrygana
Site 6, Agios Stefanos			Abandoned cultivations, phrygana
Site 7, Papalazarou fields			Phrygana, calcareous rocks
Site 8, Limenari gulf			Phrygana
Site 9, Vigla mt., around plateau			Cultivations, phrygana, calcareous rocks
Site 10, Nyftis area			Cultivations, maquis, phrygana, calcareous rocks
Site 11, Castle			Ruins, calcareous rocks
Ro isl.	1.5	100	Cultivations, maquis, phrygana, calcareous rocks
Strongyli isl.	0.93	197	Maquis, phrygana, calcareous rocks
Agios Georgios isl.	0.01	< 10	Cultivations, phrygana
Agrielia isl.	0.012	< 10	Phrygana
Psomi isl.	0.004	25	Maquis
Psoradia isl.	0.01	< 10	Phrygana, ruins

Even though, the main effort in every sampling site was focused on species richness, their abundance was recorded as dense when dozens of living individuals were observed or rare when no more than five living individuals were observed.

The collected specimens were drowned in water for 24 h and then preserved in 75% ethanol. Some of the individuals were preserved in absolute ethanol for future molecular analysis. The collected litter was sieved through 5–0.4 mm mesh and examined under a magnifying lens in the laboratory.

The above material was deposited in the NHMC collections. The present study also includes samples occasionally collected from Kastellorizo by other scientists and stored in the same collections, i.e. by University of Athens herpetologists Dr. M Polymeni and Dr. E Valakos in 1976 and 1981; by NHMC scorpiologist Dr. I Stathi in 2000; and by NHMC myriapodologist Dr. S Simaiakis in 2014.

Species identification was carried out by the authors, based on shell characteristics and reproductive system.

We also carried out cluster analysis using various similarity indices and UPGMA for the whole data set of sites and islets with the software package PAST3 [39].

## Conclusions

In total 31 species (30 extant) of terrestrial gastropods have been found on the seven studied islets. Kastellorizo Island, the largest in area and the richest as far as its snails are concerned, presents the highest diversity (86%, 19 species out of 23) in and around the main village. This could be attributed to the long history of this village, more than 2500 years.

No endemic species are present on the islets, due to their proximity and their possible connection during Late Pleistocene with the Turkish coast.

The differences in species composition and the predominant species among the three largest islets, Kastellorizo, Ro, Strongyli, can be attributed either to the paleogeography of the area as these islets were never directly connected, or to the continuous substitution from the source area.

## Data Availability

All data used or analyzed during this study are included in this published article.
